# A Wavelet-Based Approach to Fall Detection

**DOI:** 10.3390/s150511575

**Published:** 2015-05-20

**Authors:** Luca Palmerini, Fabio Bagalà, Andrea Zanetti, Jochen Klenk, Clemens Becker, Angelo Cappello

**Affiliations:** 1 Department of Electrical, Electronic, and Information Engineering “Guglielmo Marconi”, University of Bologna, 40136 Bologna, Italy; E-Mails: fabio.bagala@unibo.it (F.B.); andrea.zanetti@eggtronic.com (A.Z.); angelo.cappello@unibo.it (A.C.); 2 Department of Clinical Gerontology, Robert-Bosch-Hospital, 70376 Stuttgart, Germany; E-Mails: Jochen.Klenk@rbk.de (J.K.); Clemens.Becker@rbk.de (C.B.)

**Keywords:** fall detection, wavelet, accelerometers, pattern recognition

## Abstract

Falls among older people are a widely documented public health problem. Automatic fall detection has recently gained huge importance because it could allow for the immediate communication of falls to medical assistance. The aim of this work is to present a novel wavelet-based approach to fall detection, focusing on the impact phase and using a dataset of real-world falls. Since recorded falls result in a non-stationary signal, a wavelet transform was chosen to examine fall patterns. The idea is to consider the average fall pattern as the “prototype fall”.In order to detect falls, every acceleration signal can be compared to this prototype through wavelet analysis. The similarity of the recorded signal with the prototype fall is a feature that can be used in order to determine the difference between falls and daily activities. The discriminative ability of this feature is evaluated on real-world data. It outperforms other features that are commonly used in fall detection studies, with an Area Under the Curve of 0.918. This result suggests that the proposed wavelet-based feature is promising and future studies could use this feature (in combination with others considering different fall phases) in order to improve the performance of fall detection algorithms.

## 1. Introduction

Falls among older people are a widely documented public health problem, which can compromise quality of life and cause severe disability [1]. Around 30% of people aged 65 years or older living in the community and more than 50% of those living in residential care facilities or nursing homes fall every year, and about half of those who fall do so repeatedly [2].

Study on automatic detection of falls has become more important in last few years because it could allow for the immediate communication of falls to alert medical assistance. This automatic detection could enable rapid intervention, increasing the sense of security of older persons, and reducing some of the negative consequences of falls. The need of an accurate fall detector is hence clear.

Recent sensing hardware developments have made wearable inertial measurement units (IMUs) available for human movement analysis. Consequently, many different approaches have been explored to solve the fall detection problem using wearable IMUs, either considering dedicated wearable sensing units or using the IMU available in commercial smartphones [3,4,5,6,7]. The majority of these studies focus on the acceleration signals. The considered approaches can be divided in two main types: threshold-based and machine learning (or data mining) [6].

Both types are based on features extracted from the recorded signals; these features are usually descriptive of a certain phase of the fall. A model of the different phases of the fall was proposed in [8], dividing the fall in pre-fall, falling, impact, resting, and recovery phases.

Threshold-based methods for fall detection use single or multiple thresholds on the extracted features (e.g., if the max value of the acceleration during impact is higher than a certain threshold, then a fall is detected) [9]. On the other hand machine learning techniques use automatic methods like classifiers (e.g., Support Vector Machines) that, starting from the extracted features, try to differentiate between a fall and a normal activity of daily living (ADL) [10].

The aim of this work is to present a novel feature for fall detection, based on the wavelet-analysis of the impact phase. Wavelet transform (WT) was chosen to examine fall patterns because recorded falls result in a non-stationary signal. WT analyzes the signal at different frequencies with different resolutions. WT is therefore suitable for analyzing signals which are characterized by transient behavior or discontinuities such as falls and other transient events typical of human movement [11].

The idea of this wavelet-based approach is to consider the average fall pattern of real-world falls as the “prototype fall” (mother wavelet): in order to detect falls, every recorded acceleration signal can then be compared to this prototype through wavelet analysis. The similarity of the recorded signal with the prototype fall is the feature that can be used to discriminate between falls and activities of daily living (ADLs).

Recent reviews and surveys on fall detection using wearable sensors [3,4,5,6,7] find that the common issue to the majority of these studies is the lack of real-world falls (unintentional falls of older people), since most of these studies use simulated falls (usually performed by young people). To the best of our knowledge (and as reported in [3,4,5,6,7]), until now only five studies have evaluated the performance based on real-world falls. In Bagalà *et al.* [9], thirteen threshold-based algorithms were evaluated on a dataset of 29 real-world falls (the same one as the present study). In Tamura *et al.* [12], a threshold-based algorithm was evaluated on 22 real-world falls. In Kangas *et al.* [13], one of the threshold-based algorithms that was in [9] was evaluated on 15 real-world falls. Finally, 12 and eight real-world falls were considered in Feldwieser *et al.* [14] and Bloch *et al.* [15], respectively. In addition to these, two other studies considered real-world falls for descriptive purposes. One from Klenk *et al*. [16] which considers five backward real-world falls and the other from Kangas *et al.* [17], which considers five real-world falls, which are a subset of the falls reported in [13].

These studies have shown that algorithms tuned on simulated falls tend to underperform in real-life situations [9,13,14] and that simulated falls can show differences (although they also share common characteristics) with respect to real-world falls [16,17]. Therefore, in the present study, real-world falls and real-world ADLs are used to evaluate the performance of the proposed wavelet-based approach.

Previous applications of WT within human movement analysis have included removing noise from biomechanical data and smoothing [18], as well as classification of walking patterns and long term activity monitoring [19,20,21,22,23].

Using WT in fall detection studies based on inertial sensors has been limited. In Yavuz *et al*. [24] and Yuwono *et al*. [25], WT was used as a feature extraction method from acceleration data. The main limitation of these studies is the fact that performance is tested only on simulated falls. The present study overcomes this limitation. Moreover, the novelty of the proposed approach with respect to these studies is the use of an adapted mother wavelet (built from available fall patterns) instead of using the standard mother wavelets that are normally available. Considering different types of sensors, WT was also used on signals recorded by Doppler radar [26] and vibration sensors [27]. This highlights the wide range of possible applications of WT.

## 2. Methods

### 2.1. Real-World Falls and ADLs

**Figure 1:**
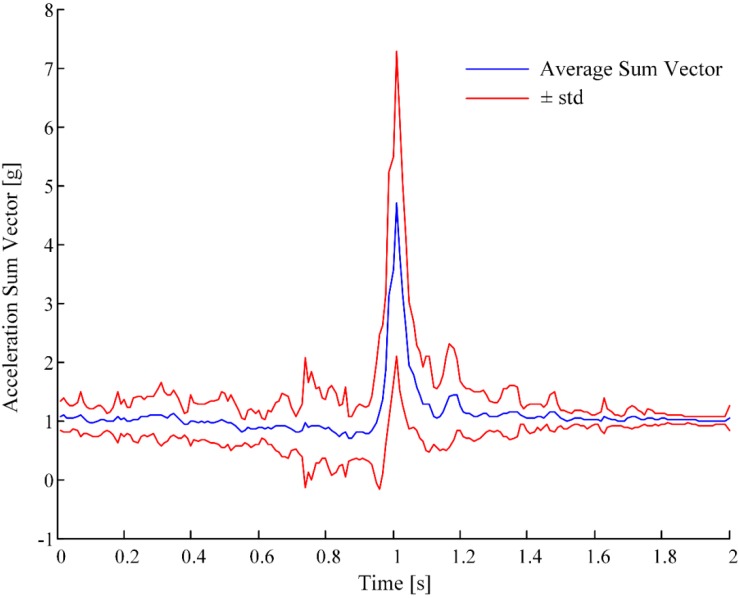
Average (±1 standard deviation) acceleration sum vector (centered on the peak) over the 29 real-world falls.

The fall database from [9], which consists of acceleration signals of 29 real-world falls from nine people, was considered. Each fall recording is one minute long, centered on the time of impact. Sixteen of these falls were recorded by the McRoberts Dynaport Minimod sensing unit from six community-dwelling patients (63 years old ± 7 years, four females) with progressive supra-nuclear palsy (PSP). Twelve of these falls were recorded by the McRoberts Dynaport Minimod Hybrid sensing unit from two PSP patients (one 71 year old female, one 72 year old male) staying in a geriatric rehabilitation unit. One fall was recorded by the McRoberts Dynaport Minimod sensing unit from one community-dwelling older person (80 year old female). We considered the acceleration signals recorded by the tri-axial acceleration sensor included in the sensing units. The acceleration sum vector (SV, root sum vector of the three squared accelerometer outputs), averaged over all the recorded falls, is shown in Figure 1.

We considered the ADL database from [9], which consists of 1170 acceleration signals (each recording is one minute long) from the PSP patients. These signals were relative to active periods that were not falls, extracted from a total of 168 hours of acceleration recordings [9]. Active periods were defined as one minute recordings where the difference between the maximum and minimum value of SV was more than 1.01 *g* (further details on this procedure can be found in [9]).

All the acceleration signals were recorded with a sampling frequency (Fs) of 100 Hz. Thirteen falls were recorded with a range of ±6 *g*. The remaining sixteen falls were recorded with a range of ±2 *g*. The ADLs were all recorded with a range of ±6 *g*. The sensing unit was held in place by a belt at the lower back of the subjects.

### 2.2. Computation of the Wavelet-Based Feature

Acceleration sum vectors (SV) of recorded falls usually exhibit an impact phase, presented as a peak, which characterizes the response to the impact (see Figure 1). Many current fall detection algorithms use a threshold on the value of this peak [9] in order to differentiate between falls and ADLs. The main idea, as presented in the introduction, is to create a prototype of typical fall pattern (mother-wavelet) by averaging the available real-world fall signals (Figure 1). Then, a degree of similarity of the signal to test with the mother-wavelet can be computed through wavelet analysis.

First, we averaged the recorded acceleration signals of real-world falls. In order to do this, we considered the 2-second window centered on the peak for each fall. These two-second windows were then averaged (as in Figure 1). The average fall pattern obtained was converted into an adapted mother wavelet, defined in the interval [0,1], and satisfying the wavelet definition [28]. The MATLAB function *pat2cwav* provided the adapted mother wavelet Ψ_fall_.

**Figure 2:**
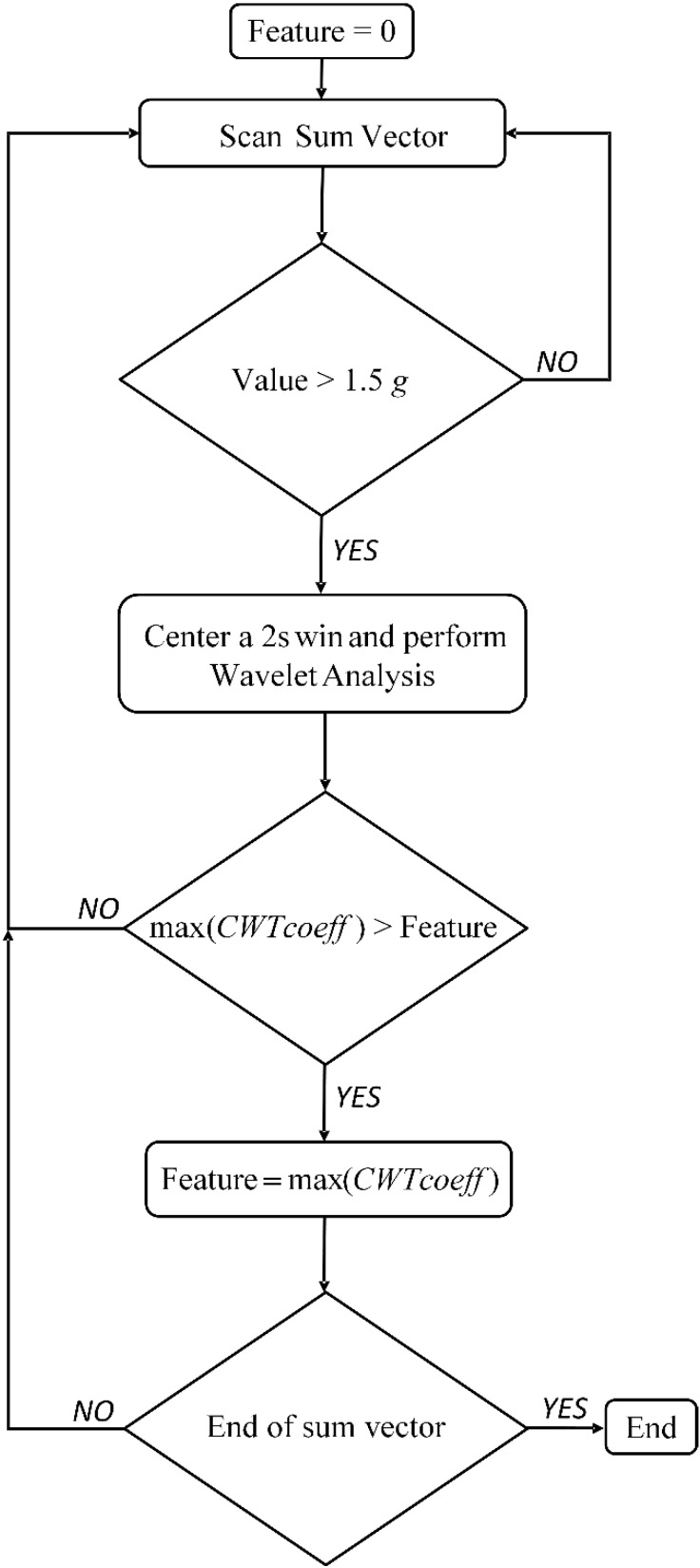
Workflow of the procedure that is used to compute the wavelet-based feature.

After the mother wavelet was created, we computed the wavelet-based feature from a recorded signal as exemplified in Figure 2. The acceleration SV of each recording (ADL or fall) was scanned, until a value greater than 1.5 *g* was found, which could represent a possible peak. This condition was set in order to reduce computational cost because wavelet analysis only starts if SV satisfies that condition. It was set to a low value so that no falls would be missed. If the condition was satisfied, then a 2-second window was centered on the corresponding sample and became the candidate window. We then compared the signal in the current window with the mother-wavelet by computing the continuous wavelet transform (CWT) coefficients (*CWTcoeff*).

These coefficients estimate the similarity of the candidate window with the mother wavelet and with scaled and translated versions of it:

(1) $$CWTcoeff\;(a,b)=\frac{1}{\sqrt{a}}\int_{-\infty }^{\infty }SV_{candidate}(t)\Psi_{fall}(\frac{t-b}{a})dt$$

In Equation (1), *a* and *b* are the scale and the translation parameters, respectively. *CWTcoeff (a,b)* describes the similarity (the higher the coefficient, the higher the similarity) between the candidate and the mother wavelet, at different scales (*a*) and translations (*b*). Then the maximum value of *CWTcoeff* was chosen.

In many contexts, it is natural to define the scales (*a*) in terms of the duration of the patterns to be detected (in this case, 2 s) instead of defining it in terms of the dilation factor. Since the mother wavelet was defined in the normalized interval [0,1] and it was generated from a pattern of 2 s, the maximum coefficient would be at a scale *a* = 2 Fs, if CWT is applied to a signal with the same shape of the mother wavelet. According to these considerations, the search for the maximum CWT coefficient was limited to scales in an interval centered on 2 Fs: 2 Fs − 0.5 Fs ≤ a ≤ 2 Fs + 0.5 Fs.

Moreover, since the maximum *CWTcoeff* values are expected to be near the center of the window (because both the mother wavelet and the candidate window are centered on the peak) the CWT coefficients with translations (*b*) smaller than 0.5 Fs or greater than 1.5 Fs were not considered. This was done in order to avoid possible border effects.

Then, the scanning of the signal continued. Each time SV was over the threshold, the analysis was repeated. In the end, the feature value was the overall maximum value of *CWTcoeff*. If no SV value over the threshold was found in the recording, then the feature value was zero (see Figure 2).

The wavelet-based feature is a continuous score which is greater than or equal to zero. The higher the score value, the higher the similarity to the mother wavelet and the probability that the recorded signal is a fall.

### 2.3. Performance Evaluation

Since the mother wavelet is created by averaging the available fall patterns, 10-Fold-cross validation was performed on the data, in order to obtain realistic results on the discriminative ability of the proposed feature (*i.e.*, the ability to differentiate between a fall and an ADL). At each step, 90% of the data (90% of fall signals and 90% of ADL signals) was randomly selected for training (the mother wavelet was constructed by averaging the fall signals in the training set) and the remaining 10% of data was used for testing.

We computed the Receiver Operating Characteristic (ROC) curve and the corresponding Area under the Curve (AUC) from the cross-validated feature values to evaluate the discriminative ability. We considered the vertical averaging methodology presented in [29] to average the cross-validated ROCs: vertical averaging takes vertical samples of the ROC curves for fixed values of specificity (from 0 to 1 with a step of 0.001) and averages the corresponding sensitivities [29].

**Figure 3:**
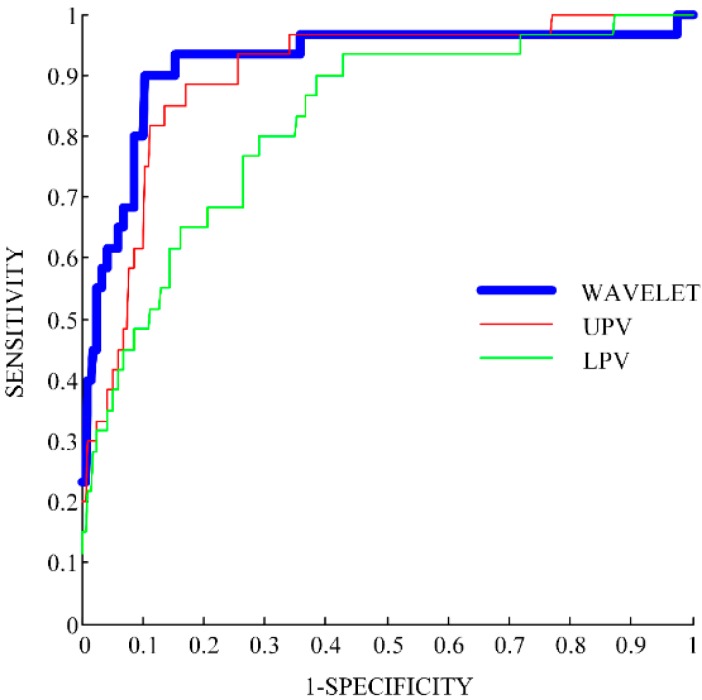
ROC curve of three features: the Wavelet-based (in blue), the Upper Peak Value (UPV, in red), and the Lower Peak Value (LPV, in green).

As in [9], we considered sensitivity as the percentage of correctly detected falls, and specificity as the percentage of correctly detected ADLs. We obtained the ROC curve as follows: after the procedure explained in Section 2.2, all falls and all ADLs have a corresponding value of the wavelet-based feature. Generally, falls tend to have higher values (since they generally are more similar to the average fall pattern) and ADLs tend to have lower values. If we set a certain threshold, then all the recordings with a value over that threshold would be detected as falls while all the recordings with a value under that threshold would be detected as ADLs. By varying this threshold, we obtained different combinations of sensitivity and specificity which corresponded to different points in ROC curves in Figure 3.

We chose the Youden’s Index (YI) criterion [30] in order to select the optimal combination of sensitivity and specificity (the optimal point in the ROC curve) for the wavelet-based feature. YI is defined as “sensitivity+specificity-1”. The optimal combination is the one with the highest value of this index and corresponds to a certain threshold on the wavelet-based feature.

We compared the performance of the wavelet-based feature (ROC, AUC, and maximum YI) with the performance of other features. In particular, we chose two features that are extracted from the impact phase and that were used in the best algorithms evaluated on real-world falls in Bagalà *et al.* [9]. These two features are the Upper Peak Value (UPV) and the Lower Peak Value (LPV). They were introduced by observing that in the typical pattern of a fall (see Figure 1), there is a phase where the acceleration SV decreases (falling phase) until reaching a minimum on the impact (LPV). This is followed by a positive peak (with a maximum value UPV), which is a response to the impact (see Figure 1) [8,9,31].

To compare the AUC and maximum YI values of the proposed feature with the AUC and maximum YI values of the other two features, paired Wilcoxon signed-rank tests (with Bonferroni correction for multiple comparisons) were performed.

### 2.4. Software

We used MATLAB R2013b for all the analyses in this study. We used the MATLAB function *perfcurve* to compute the ROC curve and the function *signrank* to perform the paired Wilcoxon signed-rank test.

## 3. Results and Discussion

The ROC curve of the proposed wavelet-based feature is presented in Figure 3.

The wavelet-based feature shows a higher ROC curve than the other features (LPC and UPV) for most of the possible combinations of sensitivity and specificity (Figure 3). In Table 1, two performance measures are reported: the AUC and the maximum YI values.

**Table 1:** Performance of the three presented features. Area under the Curve and maximum Youden’s Index values are reported with 95% Confidence Intervals. In the last two columns the p-values of the comparisons between features are reported. * = statistical significant difference (considering the Bonferroni correction for multiple comparisons).

	Wavelet	UPV	LPV	p VS UPV	p VS LPV
AUC [95% CI]	0.918 [0.848 0.99]	0.898 [0.848 0.949]	0.821 [0.724 0.919]	0.152	0.002 *
max YI [95% CI]	0.797 [0.697 0.897]	0.713 [0.613 0.813]	0.515 [0.45 0.58]	0.002 *	0.0078 *

AUC: Area Under the Curve, YI: Youden’s Index, CI: 95% Confidence Intervals.

The AUC value of the proposed feature is 0.918, significantly higher (and therefore better) than the AUC of LPV (0.821). The AUC of the proposed feature is also higher with respect to the AUC of UPV (0.898), but this difference does not reach statistical significance. In fact, it can be noted that in this case the confidence intervals are broad (because of the small sample size) and overlapping.

The maximum YI value of the wavelet-based feature (0.797) is significantly higher (and therefore better) than both the UPV (0.713) and LPV (0.515) features. AUC and max YI value are performance measures which evaluate different aspects. AUC is a global indicator (it considers all points of the ROC curve) of performance. Maximum YI on the other hand is an indicator of how the feature performs in a single point in the ROC curve (corresponding to the best combination of sensitivity and specificity).

Considering the global performance indicator (AUC), the wavelet-based features significantly outperforms only the LPV feature. However, considering the single best possible combination of sensitivity and specificity (maximum YI), the wavelet-based feature significantly outperforms both UPV and LPV. The combinations of sensitivity and specificity corresponding to the maximum YI value for all the three features are reported in Table 2. The wavelet-based feature shows a very high sensitivity (90%), while maintaining a high specificity (89.7%). The obtained specificity of 89.7% corresponds to 10.3% of false positive rate. Considering the 1170 ADL recordings, 120.5 false alarms were found. Considering that the ADLs were extracted from a total of 168 h of recording, we can estimate 0.72 false alarms/hour. So, the best combination of the proposed feature is 90% sensitivity and 0.72 false alarms/hour.

**Table 2:** Combination of sensitivity and specificity with the maximum Youden’s Index and corresponding threshold for the three features.

	Sensitivity	Specificity	Threshold
Wavelet	90%	89.7%	31.3
UPV	85%	86.3%	2.79 *g*
LPV	90%	61.5%	0.5 *g*

As shown in Table 2, UPV shows lower sensitivity (85%) and lower specificity (86.3%, corresponding to 0.95 false alarms/hour) with respect to the wavelet-based feature. LPV shows the same value of sensitivity (90%) and a very low value of specificity (61.5%, corresponding to 2.68 false alarms/hour).

If we considered the fall detection algorithms that were tested on this same dataset in a previous work [9], their false alarm rates would range from 0.14 to 6.2 false alarms/hour. Although a subset of these algorithms has a lower false alarm rate than our proposed approach, none of these has a corresponding sensitivity of 90%, as our approach does. Moreover, these algorithms also consider the resting phase in order to improve their specificity and consequently their false alarm rate.

In conclusion, the proposed wavelet-based feature shows a combination of sensitivity and specificity that outperforms impact-based features that are commonly used in fall detection studies. However, due to the high number of false alarms, it cannot be used alone to obtain an effective real-life fall detector. The proposed feature should be combined through multiple thresholds or machine learning techniques, with features extracted from other fall phases in order to improve the overall fall detection performance. Examining resting and recovery phases could be critical in order to reduce false alarms.

The main limitations of this study are the small sample size and the fact that the recorded real-world falls were mainly from a rare disease population (PSP subjects). It should be tested whether the proposed approach can be successfully generalized to the older population. The future availability of additional real-world falls from the database that is being collected during the FARSEEING [32] European project will help to overcome these limitations.

## 4. Conclusions

We have designed and implemented a novel wavelet-based approach for fall detection. It focuses on the impact phase of a fall. It is one of the first approaches that uses the fall patterns from real-world data. This is also one of the first studies using wavelet analysis for fall detection with wearable sensors ([24,25]) and the first one which builds an adapted mother wavelet using the average fall pattern of available real-world falls instead of using the standard mother wavelets [28].

In this work we present a promising feature which outperforms others that are commonly used in fall detection studies. This feature could be used in future works by combining it with features extracted from other phases of the fall. This could increase the performance of fall detection algorithms, therefore assuring effective solutions for the everyday life of older people.

## References

[1] Fuller G.F. (2000). Falls in the elderly. Am. Fam. Phys..

[2] Kannus P., Sievänen H., Palvanen M., Järvinen T., Parkkari J. (2005). Prevention of falls and consequent injuries in elderly people. Lancet.

[3] Igual R., Medrano C., Plaza I. (2013). Challenges, issues and trends in fall detection systems. Biomed. Eng. Online.

[4] Chaudhuri S., Thompson H., Demiris G. (2014). Fall detection devices and their use with older adults: A systematic review. J. Geriatr. Phys. Ther..

[5] Schwickert L., Becker C., Lindemann U., Maréchal C., Bourke A., Chiari L., Helbostad J.L., Zijlstra W., Aminian K., Todd C. (2013). Fall detection with body-worn sensors: A systematic review. Z. Gerontol. Geriatr..

[6] Pannurat N., Thiemjarus S., Nantajeewarawat E. (2014). Automatic fall monitoring: A review. Sensors.

[7] Habib M.A., Mohktar M.S., Kamaruzzaman S.B., Lim K.S., Pin T.M., Ibrahim F. (2014). Smartphone-based solutions for fall detection and prevention: Challenges and open issues. Sensors.

[8] Becker C., Schwickert L., Mellone S., Bagalà F., Chiari L., Helbostad J.L., Zijlstra W., Aminian K., Bourke A., Todd C. (2012). Proposal for a multiphase fall model based on real-world fall recordings with body-fixed sensors. Z. Gerontol. Geriatr..

[9] Bagalà F., Becker C., Cappello A., Chiari L., Aminian K., Hausdorff J.M., Zijlstra W., Klenk J. (2012). Evaluation of Accelerometer-Based Fall Detection Algorithms on Real-World Falls. PLoS ONE.

[10] Özdemir A.T., Barshan B. (2014). Detecting falls with wearable sensors using machine learning techniques. Sensors.

[11] Najafi B., Aminian K., Paraschiv-ionescu A., Loew F., Büla C.J., Robert P., Member S. (2003). Ambulatory System for Human Motion Analysis Using a Kinematic Sensor: Monitoring of Daily Physical Activity in the Elderly. IEEE Trans. Biomed. Eng..

[12] Tamura T. Wearable accelerometer in clinical use. Proceedings of the 27th Annual International Conference of the Engineering in Medicine and Biology Society, IEEE-EMBS 2005.

[13] Kangas M., Korpelainen R., Vikman I., Nyberg L., Jämsä T. (2014). Sensitivity and False Alarm Rate of a Fall Sensor in Long-Term Fall Detection in the Elderly. Gerontology.

[14] Feldwieser F., Gietzelt M., Goevercin M., Marschollek M., Meis M., Winkelbach S., Wolf K.H., Spehr J., Steinhagen-Thiessen E. (2014). Multimodal sensor-based fall detection within the domestic environment of elderly people. Z. Gerontol. Geriatr..

[15] Bloch F., Gautier V., Noury N., Lundy J.-E., Poujaud J., Claessens Y.-E., Rigaud A.-S. (2011). Evaluation under real-life conditions of a stand-alone fall detector for the elderly subjects. Ann. Phys. Rehabil. Med..

[16] Klenk J., Becker C., Lieken F., Nicolai S., Maetzler W., Alt W., Zijlstra W., Hausdorff J.M., van Lummel R.C., Chiari L. (2011). Comparison of acceleration signals of simulated and real-world backward falls. Med. Eng. Phys..

[17] Kangas M., Vikman I., Nyberg L., Korpelainen R., Lindblom J., Jämsä T. (2012). Comparison of real-life accidental falls in older people with experimental falls in middle-aged test subjects. Gait Posture.

[18] Wachowiak M.P., Rash G.S., Quesada P.M., Desoky A.H. (2000). Wavelet-based noise removal for biomechanical signals: A comparative study. IEEE Trans. Biomed. Eng..

[19] Preece S.J., Goulermas J.Y., Kenney L.P.J., Howard D. (2009). A comparison of feature extraction methods for the classification of dynamic activities from accelerometer data. IEEE Trans. Biomed. Eng..

[20] Godfrey A., Conway R., Leonard M., Meagher D., Olaighin G.M. (2009). A continuous wavelet transform and classification method for delirium motoric subtyping. IEEE Trans. Neural Syst. Rehabil. Eng..

[21] Wang N., Ambikairajah E., Lovell N.H., Celler B.G. Accelerometry based classification of walking patterns using time-frequency analysis. Proceedings of the 29th Annual International Conference of the IEEE Engineering in Medicine and Biology Society, EMBS 2007.

[22] Sekine M., Tamura T., Akay M., Fujimoto T., Togawa T., Fukui Y. (2002). Discrimination of walking patterns using wavelet-based fractal analysis. IEEE Trans. Neural Syst. Rehabil. Eng..

[23] Nyan M.N., Tay F.E.H., Seah K.H.W., Sitoh Y.Y. (2006). Classification of gait patterns in the time-frequency domain. J. Biomech..

[24] Yavuz G., Kocak M., Ergun G., Alemdar H., Yalcin H., Incel O., Ersoy C. A Smartphone Based Fall Detector with Online Location Support. Proceedings of PhoneSense 2010.

[25] Yuwono M., Moulton B.D., Su S.W., Celler B.G., Nguyen H.T. (2012). Unsupervised machine-learning method for improving the performance of ambulatory fall-detection systems. Biomed. Eng. Online.

[26] Su B.Y., Ho K.C., Rantz M.J., Skubic M. (2015). Doppler radar fall activity detection using the wavelet transform. IEEE Trans. Biomed. Eng..

[27] Yazar A., Keskin F., Töreyin B.U., Çetin A.E. (2013). Fall detection using single-tree complex wavelet transform. Pattern Recognit. Lett..

[28] Misiti M., Misiti Y., Oppenheim G., Poggi J.-M. (2007). Wavelets and Their Applications.

[29] Fawcett T. (2006). An introduction to ROC analysis. Pattern Recognit. Lett..

[30] Youden W.J. (1950). Index for rating diagnostic tests. Cancer.

[31] Bourke A.K., van de Ven P., Gamble M., O’Connor R., Murphy K., Bogan E., McQuade E., Finucane P., Olaighin G., Nelson J. (2010). Evaluation of waist-mounted tri-axial accelerometer based fall-detection algorithms during scripted and continuous unscripted activities. J. Biomech..

[32] FARSEEING. http://farseeingresearch.eu/.

